# Corrigendum to “Treatment patterns in essential tremor: Real‐world evidence from a United Kingdom and France primary care database”

**DOI:** 10.1111/ene.16218

**Published:** 2024-02-07

Antonazzo IC, Rozza D, Conti S, et al. Treatment patterns in essential tremor: Real‐world evidence from a United Kingdom and France primary care database. *Eur J Neurol*. 2024;31:e16064. doi:10.1111/ene.16064

The affiliations of the co‐authors Paolo Angelo Cortesi and Lorenzo Giovanni Mantovani was published as:

Paolo Angelo Cortesi^1^, Lorenzo Giovanni Mantovani^1^

^1^Research Centre on Public Health (CESP), University of Milano‐Bicocca, Monza, Italy

The affiliations should be revised as follows:

Paolo Angelo Cortesi^1,3^, Lorenzo Giovanni Mantovani^1,3^

^1^Research Centre on Public Health (CESP), University of Milano‐Bicocca, Monza, Italy

^3^Istituto Auxologico Italiano ‐ IRCCS, Milan, Italy

We would like to apologize for any inconvenience or misunderstanding.

